# Altered Salivary Microbiota Following *Bifidobacterium animalis* Subsp. *Lactis* BL-11 Supplementation Are Associated with Anthropometric Growth and Social Behavior Severity in Individuals with Prader-Willi Syndrome

**DOI:** 10.1007/s12602-022-09938-0

**Published:** 2022-04-26

**Authors:** Kevin Liu, Xue-Jun Kong

**Affiliations:** 1 Athinoula A. Martinos Center for Biomedical Imaging, Massachusetts General Hospital, 149 13th Street, Charlestown, Boston, MA 02129 USA; 2 Department of Medicine and Psychiatry, Beth Israel Deaconess Medical Center, Boston, MA 02215 USA

**Keywords:** Prader-Willi syndrome, Saliva, Microbiome, Probiotics, *Bifidobacterium*, Height

## Abstract

To evaluate the influence of oral probiotic *Bifidobacterium animalis* subsp. *lactis* (BL-11) supplementation on salivary microbiota composition and the association with growth parameters, and behavioral symptoms in individuals with Prader-Willi syndrome (PWS). In this post hoc analysis, we included a subset of 36 PWS patients with available saliva samples from our original randomized, double-blinded, placebo-controlled trial (Chinese Clinical Trial Registry, ChiCTR1900022646, April 20, 2019). Among the 36 subjects, 17 subjects were allocated to the probiotic group for daily use of the BL-11 probiotic and 19 subjects were allocated to the placebo group. Groupwise and longitudinal differences in salivary microbiota abundances, biodiversity metrics, and height were analyzed. Linear correlations were found between identified differentially abundant salivary microbiota and clinical parameters. Salivary microbiome α-diversity was found to be higher in the probiotic-treated group at week 12 relative to placebo controls (*P* < 0.05). *Leptotrichia*, *Paracoccus*, and *Faecalibacterium* were found to be more abundant in the probiotic-treated group (*P* < 0.05). Salivary microbiota abundance and predicted functional profiling abundance correlations were found to be associated with anti-inflammation, anti-obesity, toxin degradation, and anti-oxidative injury effects (*Q* < 0.1). Several oral taxa also displayed correlations with social behavior severity scores in the probiotic-treated group (*Q* < 0.1). The findings suggest novel salivary microbiota compositional changes in response to the oral supplementation of BL-11 probiotic in individuals with PWS. The observed differentially abundant taxa between groups post-treatment were highly correlated with interventional effects on growth and social behaviors, although further investigation is warranted. *Clinical Trial Registration* The original clinical trial was registered under the Chinese Clinical Trial Registry with registration number ChiCTR1900022646 (April 20, 2019).

**Supplementary Information:**

The online version contains supplementary material available at 10.1007/s12602-022-09938-0.

## Introduction

Prader-Willi syndrome (PWS) is a rare genetic disorder featuring severe hypotonia and feeding difficulties in early infancy and subsequent hyperphagia and early childhood onset obesity. Additionally, developmental delay, short stature, and numerous neuropsychiatric comorbidities have been implicated in individuals with PWS and it has been recognized as a type of syndromic autism spectrum disorder (ASD) [1–4]. Given the diverse array of symptoms associated with PWS and an increasing popularity in multi-omics research, there is a growing body of literature supporting linkages between alterations in the gut microbiota and the clinical manifestations of PWS-associated features; however, few studies have attempted to characterize the salivary microbiome composition and elucidate the potential interactions with the gut microbiome. Specifically, while past studies have shown that dysbiotic gut microbiota of individuals with PWS have been implicated in the etiology of obese PWS patients and associated worsening of insulin tolerance [5, 6], the host-salivary microbiota interactions and potential salivary microbiome dysbiosis in PWS patients have yet to be explored despite evidence found within individuals with ASD [7, 8]. As several past studies have shown improvements in metabolic disturbances and gut microbiome dysbiosis in both obese mice and overweight human adults following probiotic supplementation [9, 10], we recently published two double blinded, randomized, and placebo-controlled clinical trials of probiotic supplementation for the treatment of anthropometric growth-associated comorbidities in PWS [11, 12]. In our study on *Bifidobacterium animalis* subsp*. lactis* (BL-11) supplementation in individuals with PWS, we found a significant increase in height and improvement in behavioral symptoms following probiotic treatment and both the gut microbiota composition and metagenomic functional profiles also favored effects of weight loss and gut health with increased abundances of antioxidant production-related genes [12]. While such linkages have been established between changes in gut microbiota and the developmental features of PWS in our past studies, associated changes in the oral microbiome have yet to be elucidated despite the known interactions between the salivary and gut microbiomes in ASD [13]; thus, we hypothesize that probiotics targeted at modulating the gut microbiota may also display altered patterns of oral flora.

In contrast to the well-studied gut microbiome, characterization of salivary microbiota composition and biodiversity have not yet been explored in PWS populations despite that the oral microbiome has been recognized as potential key biomarkers of several oral and systemic diseases that may be related to symptoms of PWS [14]. For example, PWS patients were found to have high rate of oral diseases such as caries and tooth wear due to developmental delay, hyperphagia, and thick saliva [15]. As the human salivary microbiota is comprised of highly diverse groups of commensal, symbiotic, and pathogenic microorganisms, current research has suggested that such influences of the salivary microbiota extend beyond that of the oral cavity [16]. For instance, existing literature have shown that oral microbiota possesses the ability to translocate to the gut with the potential to modulate the gut microbiome, host immune defense, and brain function [17, 18]. Since our probiotic is supplied in a powder-containing sachet format, we believe that the BL-11 probiotic supplementation has the ability to influence the oral microbiome in addition to modulating the gut microbiome given that the active probiotic powder passes through the oral cavity.

With our current understanding of the multidimensional interactions of salivary microbiota with gut microbiota, immune function, and brain function, it is of interest to study such associations within the context of individuals with PWS. However, these areas of interests regarding the salivary microbiome, its relationship with core symptoms of PWS and gut microbiome composition, effects of longitudinal probiotic supplementation, and subsequent changes in the salivary microbiota in individuals with PWS have not been previously explored. To fill these gaps of knowledge on the salivary microbiota in PWS, we performed the present post hoc analysis based on our recently published clinical trial [12] to characterize the oral microbiome profile in PWS patients, examine its changes following BL-11 probiotic intervention, and assess its associations with height growth, social behavior symptom severity, and the relative levels of metagenomic functional pathways.

## Materials and Methods

### Study Design

**Fig. 1:**
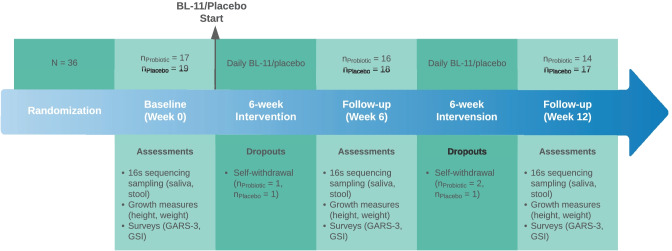
Timeline of the present study and associated participant dropouts

The original clinical trial design, protocol, randomization, blinding, participant eligibility, and intervention were well described in our previous publications [11, 12]. The original clinical trial was registered under the Chinese Clinical Trial Registry with registration number ChiCTR1900022646 (April 20, 2019) and involved 65 PWS subjects that were double-blinded and randomly assigned to either the probiotic interventional group or the placebo control group [12]. The enrolled subjects were subject to treatment for a total duration of 12 weeks. In this post hoc analysis study, we included a subset of 36 subjects with ages 59.49 ± 40.56 months who had available salivary sample 16 s sequencing data. Among the subset of 36 subjects, 17 subjects (aged 60.66 ± 32.19 months) were allocated to the probiotic group and 19 subjects (aged 58.5 ± 47.34 months) were allocated to the placebo group. The probiotic *Bifidobacterium Animalis* subsp. *lactis* BL-11 (Beijing Huayuan Academy of Biotechnology) was used in the study in the format of a sachet containing the probiotic BL-11 in powder form. Each sachet of probiotics supplement contained 3 × 10^10^ colony forming units (CFUs). The placebo was maltodextrin in the sachet with similar color, flavor, and taste as the probiotic sachets. Subjects received one sachet twice a day of either probiotic or placebo for a duration of 12 weeks and were instructed to consume the sachet contents orally with water. No adverse events were observed throughout the study course. An illustration of the timeline, sample sizes, and participant dropouts is shown in Fig. 1.

### Outcome Measurements and Data Collection

Outcome measurements were taken at weeks 0 (baseline), 6, and 12. Weight and height measurements were measured by parents using standard scales and recorded by the research staff for all enrolled subjects regardless of age. Restricted/repetitive behaviors (RRB), social interaction (SI), social communication (SC), emotional response (ER), cognitive style (CS), and maladaptive speech (MS) were evaluated by an experienced clinician via the Gilliam Autism Rating Scale, Third Edition (GARS-3), for those of ages 3 years or older [19]. Furthermore, medical, dental, and dietary histories were recorded during the visits.

### DNA Extraction and 16S Ribosomal RNA Amplicon Sequencing of Saliva Samples

Bacterial genomic DNA was extracted from saliva samples using the Powersoil DNA isolation kit (Qiagen, Duesseldorf, Hilden, Germany) with the bead-beating method according to the manufacturer’s instructions. Characterization of the salivary microbiota was done via high fidelity 16S rRNA amplicon gene sequencing based on collected saliva samples of all subjects of this study.

### Bioinformatics Processing of Amplicon Sequencing Data

Sequencing reads were bioinformatically processed using Biobakery Workflows (v0.13.2) [20] based on the VSEARCH (v2.14.1) [21] method. In short, the sequences were demultiplexed and VSEARCH was used with default parameters to merge, filter, and trim the Illumina data. The sequences were then dereplicated, sorted by size, and clustered into operational taxonomic units (OTUs). Next, phylogenetic trees were constructed using FastTree [22] after alignment of sequences using Clustal Omega [23]. The taxonomies of OTUs were assigned using the Greengenes database (v13.8) with sequences sharing 97% similarity [24]. Both R and the R package phyloseq [25] were used to perform downstream metagenomic processing, including agglomeration of OTUs at the genus level, transformation of reads to relative abundances, OTU filtering using a prevalence threshold of 0.0001 and an occurrence threshold of 10% of the population, and calculation of α-diversity indices. For creating co-abundance networks via MetagenoNets [26], reads were transformed via total sum scaling and filtered using a prevalence threshold of 0.0001 and an occurrence threshold of 10% of the population.

### Statistical Analysis

All raw data were recorded and processed in Microsoft Excel 2016 and R. Statistical procedures were carried out using *α* = 0.05 as the significance level and Q < 0.1 for adjusted *P*-values. Data analysis and visualization were performed under R using the tidyverse packages while statistics were generated using the compatible ggpubr and rstatix package. Linear mixed effects models and associated analysis were performed using the lme4 and emmeans packages.

Linear mixed effects models with maximum likelihood estimation were used to test the impact of BL-11 probiotic supplementation on the course of outcomes from the three study timepoints (weeks 0, 6, and 12) compared to receiving the placebo. In the models, treatment group (probiotic and placebo groups), study timepoint (weeks 0, 6, and 12), and the interaction between treatment group and timepoint were set as fixed effects. Participants were included as random effects. The effect of BL-11 probiotic supplementation was estimated based on the interaction between treatment group and timepoint. Furthermore, the post hoc interaction contrasts between treatment group and timepoint were determined.

Differential abundance analysis at the genus level was conducted using linear discriminant analysis (LDA) effect size (LEfSe) [27] by applying an LDA score minimum threshold of 2 and a significance cutoff of *α* = 0.05 for groupwise comparisons. Univariate linear correlations via MaAsLin2 [28] were used to explore per-feature correlations between clinical indices, including GARS-3 total and subscale scores, weight, height, and BMI, predicted functional profiling features, and genus-level OTU abundances; the resulting *P*-values were adjusted for multiple testing using false-discovery rate (FDR). Genus-level salivary microbiota co-abundance network analyses were processed, analyzed, and plotted in MetagenoNets [26] using the NAMAP with Spearman’s rank correlation algorithm while applying a significance cutoff of *α* = 0.05 with 100 bootstrap iterations.

## Results

### Summary of Baseline Subject Demographics and Clinical Indices

**Fig. 2:**
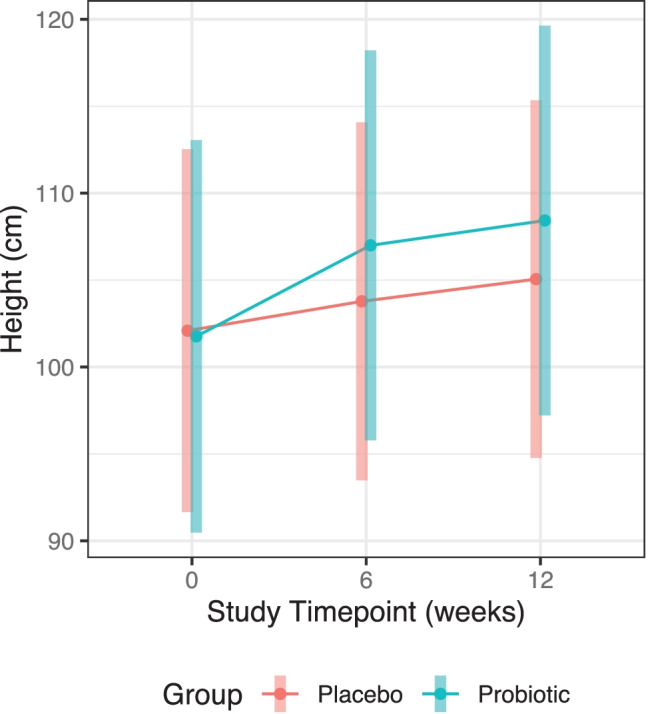
Estimated marginal means for the linear mixed effects model of height per timepoint by group with 95% confidence intervals

We included 36 subjects aged 59.49 ± 40.56 months (52.78% male, 47.22% female) with genetically confirmed diagnosis of PWS. Of which, 17 subjects aged 60.66 ± 32.19 months were randomized to receive the active probiotic, while 19 subjects aged 58.5 ± 47.34 months were randomized to receive placebo for a duration of 12 weeks. Among the 36 subjects included in this study, there were 29 of them had available GARS-3 dataset (GARS-3 is only applicable for those age 3 or above), 17 subjects were in placebo group, and 12 subjects were in probiotics group. No adverse events were reported during the trial period. A summary of the subject demographics and detailed clinical indices are provided in Table 1, which indicated no significant differences between the probiotic and placebo groups among all the demographic and clinical parameters as listed. We analyzed and observed a trend of increasing height over time in those receiving the probiotic treatment, but not in those receiving the placebo (Fig. 2). We performed a linear mixed effects model analysis to determine the effects of treatment groups over the three study timepoints for height. The estimated marginal means (EMM), their standard errors (SE), and the contrasts between the three study timepoints for height are detailed in Table 2, accompanied by the differences in the contrasts between the treatment groups and timepoints. Of note, the comparisons using a series of contrasts have shown significant differences for the differences in height in the probiotic group between weeks 12 and 0 and weeks 6 and 0 (Table 2, *P* < 0.001).

**Table 1:** Summary of baseline subject demographics and measured clinical indices

	**Placebo (*n* = 19)**		**Probiotic (*n* = 17)**		***χ***^**2**^**-test *P*-value**	**Overall (*n* = 36)**	
Sex (*n*)							
Male	10 (52.63%)		9 (52.94%)		0.99	19 (52.78%)	
Female	9 (47.37%)		8 (47.06%)			17 (47.22%)	
	**Mean (SD)**	**Median (IQR)**	**Mean (SD)**	**Median (IQR)**	**Mann–Whitney *U* test *P*-value**	**Mean (SD)**	**Median (IQR)**
Age (month)	58.5 (47.34)	40.5 (53)	60.66 (32.19)	58.5 (43.38)	0.48	59.49 (40.56)	46.5 (48.25)
Height (cm)	111.25 (20.71)	112.5 (33.25)	107.4 (21.37)	112 (33)	0.81	109.11 (19.83)	112 (37)
Weight (kg)	26.45 (15.29)	26.65 (25.35)	29.8 (20.02)	29 (24)	0.90	28.31 (17.06)	29 (24.8)
Body mass index (BMI)	19.47 (5.04)	19.55 (8.49)	23.01 (9.07)	18.86 (13.39)	0.73	21.43 (7.36)	18.86 (8.55)
GARS-3	*n* = 17		*n* = 12			*n* = 29	
Cognitive style (CS)	10 (3.61)	11 (3.5)	8.67 (2.08)	8 (2)	0.82	9.33 (2.73)	9.5 (3.75)
Emotional responses (ER)	10.33 (3.21)	9 (3)	15.33 (4.04)	13 (3.5)	0.38	12.83 (4.26)	13 (3.75)
Maladaptive speech (MS)	10 (5.29)	12 (5)	7.33 (3.79)	9 (3.5)	0.40	8.67 (4.37)	9.5 (6.25)
Restricted/Repetitive behaviors (RRB)	19 (6)	19 (6)	24.33 (9.29)	20 (8.5)	0.70	21.67 (7.58)	19.5 (5.5)
Social communication (SC)	19.33 (6.81)	17 (6.5)	12.33 (7.57)	9 (7)	0.40	15.83 (7.49)	15.5 (9.75)
Social interaction (SI)	10.67 (2.52)	11 (2.5)	9.33 (8.02)	10 (8)	1.00	10 (5.37)	10.5 (4)

**Table 2:** Summary of estimated marginal means and computed contrasts for linear mixed effects model analysis using timepoint and group interaction as fixed effects and subjects as random effects

**Group**	**Timepoint** **(weeks)**	**Contrast**	**EMM (SE)**	**95% CI**	**df**	***t*-ratio**	***P*-value**
Probiotic	12		108.43 (5.53)	(90.39, 126.47)	37.32		
		Probiotic 12–placebo 12	3.37 (7.51)	(−21.11, 27.86)	37.31	0.449	0.838
		Probiotic 12–probiotic 6	1.43 (0.59)	(−0.5, 3.35)	35.61	2.424	0.077
		Probiotic 12–placebo 6	4.65 (7.52)	(−19.85, 29.15)	37.40	0.619	0.838
		Probiotic 12–probiotic 0	6.67 (0.95)	(3.56, 9.77)	35.71	7.021	< 0.001
		Probiotic 12–placebo 0	6.34 (7.57)	(−18.28, 30.96)	38.35	0.837	0.838
Placebo	12		105.06 (5.08)	(88.5, 121.61)	37.31		
		Placebo 12–probiotic 6	−1.94 (7.51)	(−26.43, 22.54)	37.34	−0.259	0.854
		Placebo 12–placebo 6	1.28 (0.59)	(−0.65, 3.21)	35.64	2.167	0.111
		Placebo 12–probiotic 0	3.29 (7.54)	(−21.27, 27.86)	37.93	0.436	0.838
		Placebo 12–placebo 0	2.97 (1.14)	(−0.75, 6.68)	35.79	2.608	0.066
Probiotic	6		107 (5.54)	(88.95, 125.05)	37.36		
		Probiotic 6–placebo 6	3.22 (7.52)	(−21.28, 27.72)	37.43	0.429	0.838
		Probiotic 6–probiotic 0	5.24 (0.99)	(2.02, 8.46)	35.72	5.317	< 0.001
		Probiotic 6–placebo 0	4.91 (7.57)	(−19.72, 29.54)	38.38	0.649	0.838
Placebo	6		103.78 (5.09)	(87.21, 120.35)	37.50		
		Placebo 6–probiotic 0	2.02 (7.55)	(−22.56, 26.59)	38.02	0.267	0.854
		Placebo 6–placebo 0	1.69 (1.09)	(−1.89, 5.27)	35.75	1.542	0.330
Probiotic	0		101.76 (5.58)	(83.61, 119.91)	38.45		
		Probiotic 0–placebo 0	−0.33 (7.6)	(−25.03, 24.38)	38.97	−0.043	0.966
Placebo	0		102.09 (5.16)	(85.33, 118.85)	39.58		

### Salivary Microbiome Biodiversity and Co-abundance Network Changes Following BL-11 Supplementation

**Fig. 3:**
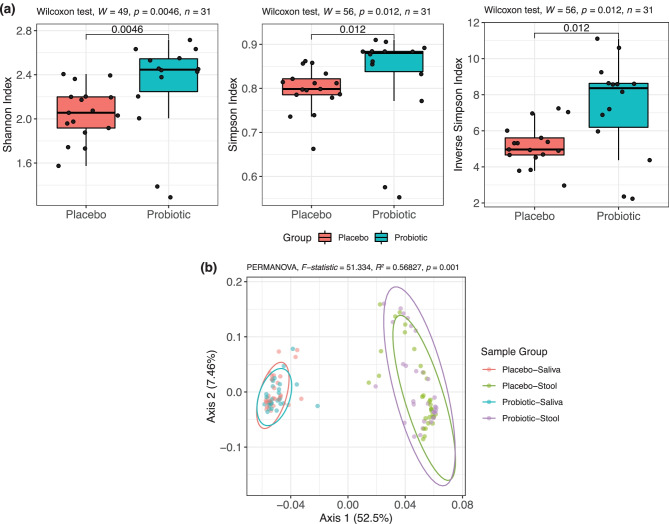
Overview of salivary and fecal microbiome biodiversity measures. **a** Genus-level α-diversity via Shannon index, Simpson index, and Inverse Simpson index at week 12 for the salivary microbiome. Groupwise comparison were conducted via Wilcoxon rank-sum test. **b** PCoA of filtered fecal and salivary genera using Bray–Curtis dissimilarity β-diversity post-treatment (i.e., weeks 6 and 12 combined). The PCoA plot explained 59.96% of the variance and 95% confidence ellipses are shown. Differences in sample groups were assessed via PERMANOVA and relevant statistics are reported above the plot

Changes in the salivary microbiome biodiversity were assessed through groupwise comparisons at each study timepoint via Mann–Whitney *U* tests for α-diversity indices at weeks 0, 6, and 12 (Online Resource 1). An increasing trend in Shannon index, Simpson index, and Inverse Simpson index was observed over the 12-week study period between groups and a significant groupwise difference is observed at week 12 (Fig. 3A, Mann–Whitney *U* test, *P* < 0.05). Bray–Curtis β-diversity was plotted using principal coordinates analysis (PCoA, Fig. 3B) and showed statistically significant differences between the four sample groups, including placebo-stool, placebo-saliva, probiotic-stool, and probiotic-saliva groups (*F* = 51.334, *R*^2^ = 0.56827, *P* = 0.001).

**Fig. 4:**
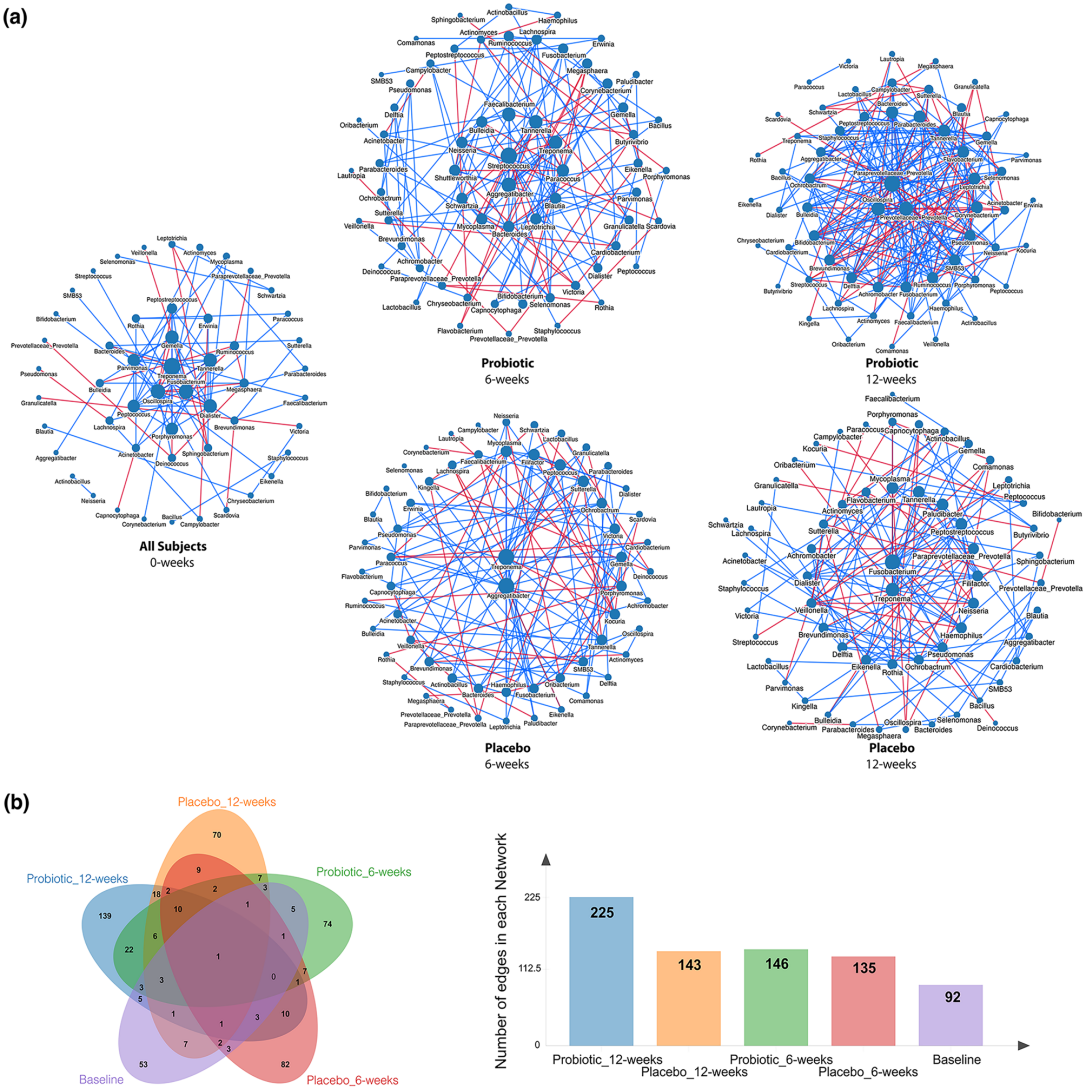
Genus-level salivary microbiota co-abundance networks by group per each study visit via the NAMAP with Spearman’s rank correlation algorithm. **a** Salivary co-abundance networks were visualized at weeks 0 (baseline, all subjects), 6, and 12 for each group. Nodes (circles) are sized and ordered concentrically by degree (i.e., number of connected edges). Edges (lines) are colored by the directionality of the correlation between two features. **b** Venn diagram of salivary taxa edges shared between groups and treatment status. Numbers in bold show the number of identified edges within the specified group and study timepoint

To characterize the interactions between genus-level salivary microbiota through their respective abundances by group per each study timepoint, we constructed co-abundance networks based on the NAMAP with Spearman’s rank correlation algorithm as shown in Fig. 4A. An overview of the number of unique and shared edges within each group based on the treatment status is shown in Fig. 4B.

### Differentially Abundant Genus-Level Salivary Microbiota Following BL-11 Supplementation

**Fig. 5:**
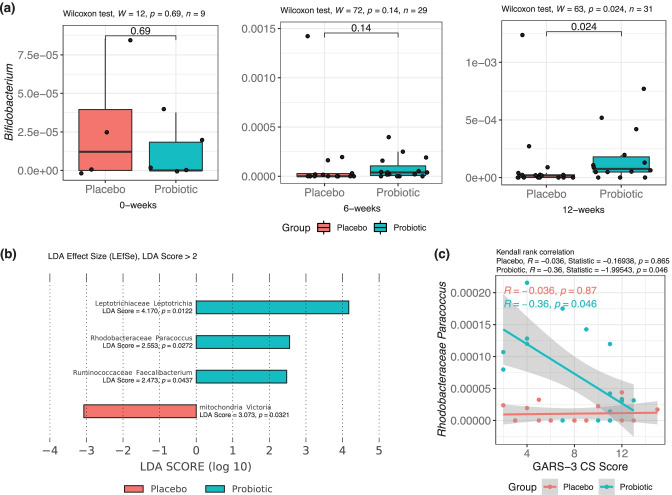
Differentially abundant salivary microbiota and associated correlates following treatment with BL-11 probiotic. **a** Salivary Bifidobacterium increases over the study period in the active probiotic group. **b** Linear discriminant analysis effect size (LEfSe) intergroup comparisons of salivary microbiota relative abundance between groups following treatment (6 and 12 weeks combined). **c** GARS-3 cognitive ability (CS) score is significantly negatively correlated with *Paracoccus* in subjects of ages 3 and older that are receiving the active probiotic following treatment (6 and 12 weeks combined)

The salivary microbiota abundance changes over the course of probiotic supplementation were assessed through both groupwise comparisons of genus-level microbiota relative abundances for specific genera of interest, including the *Bifidobacterium* genus, and via LEfSe for comparison of overall genus-level microbiota abundance changes post-treatment (i.e., samples from 6 to 12 weeks combined). The *Bifidobacterium* genus relative abundance displayed an increasing trend over the treatment course in the active probiotic group and showed significant groupwise differences in relative abundance at week 12 (Fig. 5A, Mann–Whitney *U* test, *W* = 63, *P* < 0.05). Examining all remaining genus-level OTUs following filtering by prevalence and occurrence thresholds, the *Leptotrichia* (LDA score = 4.170, *P* < 0.05), *Paracoccus* (LDA score = 2.553, *P* < 0.05), and *Faecalibacterium* (LDA score = 2.473, *P* < 0.05) genera were significantly more abundant in the probiotic group salivary microbiome, whereas the *Victoria* genus (Mitochondria) was significantly more abundant in those receiving placebo (LDA score = 3.073, *P* < 0.05, Fig. 5B). Among the three identified differentially abundant genera determined via LEfSe in the probiotic group, *Paracoccus* relative abundance was found to be negatively correlated with GARS-3 cognitive style (CS) score (Fig. 5C, Kendall rank correlation, *R* =  − 0.036, *P* < 0.05) while other differentially abundant microbiota were not found to be significantly correlated with social behavior severity scores.

### Associations Between Post-Treatment Social Behavior Severity, Height, Weight, Predicted Functional Pathways, and Salivary Microbiota Abundance

In an attempt to elucidate the functional role of differentially abundant microbiota and determine the relationships between salivary microbiota relative abundance, social behavior symptom severity, weight, and height following active probiotic supplementation, we performed univariate linear regression with FDR adjustments for genus-level microbiota correlations against height, weight, and GARS-3 total and subscale scores. Similarly, functional metagenomic profiles were correlated against the three differentially abundant microbiota identified with higher abundance in the probiotic group, including *Leptotrichia*, *Paracoccus*, and *Faecalibacterium* to explore the potential microbial functions associated with each genus. Statistical significance was considered by applying a significance cutoff of *FDR* < 0.1 and the resulting correlations are presented in the figures presented in Online Resources 2 and 3.

## Discussion

In the present post hoc study, we explored the salivary microbiota differences before and after supplementation with the probiotic *Bifidobacterium animalis* subsp. *lactis* BL-11 in individuals with PWS and assessed their associations with predicted functional profiles, behavioral severity scores, and anthropometric growth measures. Despite having a smaller sample size in this study (*N* = 36) compared to the original trial (*N* = 65) due to missing saliva 16 s rRNA amplicon sequencing samples, we were able to retain groupwise symmetry in age, height, weight, and behavioral severity parameters at baseline. Furthermore, we were able to confirm the trend of height increase with statistical significance found between weeks 12 and 0 as well as between weeks 6 and 0 in the probiotic group, which are consistent with our findings in the original trial [12]. Performed as a result of the smaller sample size in the present study, the results of these confirmatory analyses further validated our current study population as representative of the original trial.

In contrast to the well-reported gut microbiota changes with probiotic supplementation in individuals with PWS, salivary microbiota changes following probiotic intervention have not been previously reported. In this study, we found that α-diversity in terms of Shannon index, Simpson index, and Inverse Simpson index exhibits an increasing trend over the treatment course for those receiving the BL-11 probiotic and is significantly higher relative to the placebo controls at week 12. The PCoA of Bray–Curtis dissimilarity β-diversity shows defined, separated clusters between salivary and fecal microbiome clusters regardless of the treatment group (i.e., receiving either BL-11 or placebo), which is suggestive of inter-microbiome differences in biodiversity and is consistent with our expectations. Namely, we attribute the statistical significance found between the four sample groups to the compositional differences between PWS individuals’ salivary and fecal microbiomes as the distinct separation between the salivary and stool clusters are separated by axis 1, which explained 52.5% of the variance among participants, rather than axis 2 that only explained 7.46% of the variance. Of note, the evaluation of the β-diversity differences for the salivary microbiome is likely limited by the smaller sample size relative to the fecal microbiome. As opposed to our previous findings in the gut microbiome β-diversity between treatment groups [12], the results regarding the salivary microbiome biodiversity from the present study suggests that while oral supplementation of BL-11 does not alter the β-diversity between treatment groups, subjects receiving the BL-11 probiotic independently exhibit more heterogeneity in the salivary flora as shown through the higher α-diversity at week 12. To further characterize the interactions between salivary microbiota of those receiving the BL-11 probiotic relative to those receiving the placebo, we constructed genus level co-abundance networks and observed higher numbers of edges in the post-BL-11 treatment group at week 12. The larger number of edges (i.e., significant correlations) at week 12 between microbiota abundances of the probiotic group exhibits a 1.6-fold increase over that of the placebo group and 139 edges are uniquely found within the probiotic group at week 12, which suggests that introduction of the BL-11 probiotic to the oral cavity has significantly enhanced the interactions between salivary microbiota in addition to the induction of an increase in salivary microbiome α-diversity; however, the physiological implications regarding an increase in genus-level co-abundance network edges post-probiotic treatment remain largely unknown and warrant further investigation. Nonetheless, we attempt to explain the changes in the salivary microbiota composition and its host interactions through specific identified differentially abundant genera. Probing for differentially abundant microbiota following probiotic supplementation, we first analyzed the genus *Bifidobacterium* to confirm that its supplementation can be detected as an increase in the overall genus-level relative abundance. We observed an increasing trend that shows significantly higher abundances of salivary *Bifidobacterium* following BL-11 treatment relative to those receiving the placebo. The change in salivary *Bifidobacterium* relative abundance at week 12 is consistent with expectations due to the method of probiotic delivery; as the probiotic was administered orally in a powder format, we expected that exposure to the BL-11 probiotic within the oral cavity can lead to the increase in oral *Bifidobacterium* over time. Assessing for differentially abundant salivary microbiota among all identified genera, we found that subjects receiving the BL-11 probiotic intervention have higher abundances of several bacterial genera relative to those receiving the placebo; these genera include *Faecalibacterium*, *Paracoccus*, and *Leptotrichia*, and will be further discussed in terms of relevance to host growth and behavioral severity below.

Relative to host-microbiota interactions mediated via the gut-brain axis, our current understanding of the oral-gut-brain axis remains largely unknown, though recent literature has suggested that oral bacterial species and their metabolites can affect the brain either directly through the cranial nervous system and circulating blood or indirectly through gut microbiota dysbiosis and systemic inflammation [29]. Furthermore, a growing body of literature has implicated multidirectional interactions between the host immune system, gut-brain interactions, and the influence of gut microbiota on growth hormone (GH)/insulin-like growth factor 1 (IGF-1) expression [13, 17, 30, 31]. Thus, we postulate that the associations identified between salivary microbiota, height, and behavioral severity scores in the present study are driven by the probiotic-inducted compositional changes in salivary microbiota, ectopic translocation of oral microbiota to the gut, and subsequent modulation of gut microbiota to influence host growth through the GH/IGF-1 axis in individuals with PWS. In this study, we found that several salivary microbiota were significantly positively correlated with height post-BL-11 treatment, including the genera *Gemella*, *Aggregatibacter*, *Corynebacterium*, *Fusobacterium*, and *Treponema*, while such correlation were not statistically significant in those receiving the placebo. Given our current understanding, the identified height-correlated genera in the present study have been described in literature as largely non-pathogenic genera and are commonly found within the oral microbiome [14, 32, 33]. We postulate that these oral taxa may play a role in the mediation of childhood growth through ectopic transfer to the gut and subsequent interactions with gut microbiota, specifically in terms of height. In a study conducted by Vonaesch et al. the over-representation of oropharyngeal microbiota within the gut has been proposed to be associated with stunted growth in African children aged 2 to 5 years old [34]. Among the several over-represented oropharyngeal microbiota in the gut of children with stunted growth identified by Vonaesch et al. the genera *Gemella*, *Fusobacterium*, and *Aggregatibacter* were found to be positively correlated with height post-BL-11 treatment in the present study, thereby supporting our hypothesis of the oral-gut-brain axis in mediating host growth. These findings warrant further exploration of the oral-gut-brain interactions and their relationship with the phenotypes of PWS, which may facilitate the identification of new targeted therapies.

With a growing body of literature supporting the regulation of social behaviors via gut microbiota [35–37], we attempt to further assess the associations between salivary microbiota and behavioral severity in PWS under the hypothesis that alterations in oral microbiota can indirectly contribute to the modulation of behaviors via the oral-gut-brain axis. In our assessment of microbiota associations against social behavior scores, *Neisseria* was found to be positively correlated with both GARS-3 cognitive style and maladaptive speech scores while *Germella* was found to be positively correlated with only maladaptive speech score following BL-11 supplementation. Provided that we have previously found compositional differences in both salivary and fecal microbiota between individuals with autism spectrum disorder (ASD) and healthy controls [13], recent literature has further identified associations between several mental disorders and oral microbiota dysbiosis [38], thereby supporting our hypothesis of oral-gut-brain interactions in PWS. Furthermore, among the three microbiota that are enriched among the salivary microbiota, the species of the *Paracoccus* genus also presents its relevance to host behavioral alterations. The *Paracoccus* genus has been previously identified in the skin flora of healthy individuals [39], which may implicate aberrant behavioral patterns in PWS as a potential cause of the inhabitance of such microbiota within the salivary microbiome. Interestingly, we identified a significant negative correlation between the relative abundance of a *Paracoccus* species and GARS-3 cognitive style score among subjects receiving the BL-11 probiotic while this trend was not found to be statistically significant among subjects receiving the placebo. We postulate that supplementation with the BL-11 probiotic induced an increase in the abundance of *Paracoccus* identified within the salivary microbiota, which could drive the release of oxytocin (OT) or other neurotransmitters through the proposed oral-gut-brain axis, similar to the previously reported effects of *Limosilactobacillus reuteri* probiotic-induced OT and GH release [40, 41]. OT is a well-known critical in modulating emotional and social communication [42]. Additionally, we found that the *Paracoccus* species were positively correlated with caffeine metabolism in those receiving the BL-11 probiotic. Caffeine was found to help fat utilization and reduction of obesity [43, 44], which is another hallmark feature of PWS. Taken together, the associations between the relative abundance of *Paracoccus* genus against behavioral severity scores and caffeine metabolism may implicate the salivary *Paracoccus* genus as a biomarker for the presence of social behavior deficits, co-morbid ASD symptoms, and probiotic treatment response if confirmed by future large-scale studies.

Among the three genera found to be enriched in PWS subjects receiving the probiotic treatment, both *Faecalibacterium* and *Leptotrichia* present relevance to host immunity and inflammatory responses. *Faecalibacterium prausnitzii* as the sole known species belonging to the *Faecalibacterium* genus was observed to have higher abundances post-probiotic treatment, which may mediate host immune function through its butyrogenic effects within the gut microbiome [45]. A growing body of literature has suggested that the production of short chain fatty acids (SCFAs) within the gut possess anti-inflammatory effects, with acetate, propionate, and butyrate being the most abundant products [45, 46]. Mechanistic studies have demonstrated SCFA activation of mammalian G protein-coupled receptors (GPCRs) GPR41 and GPR43 *in* vitro [47, 48]. Furthermore, studies in mice have suggested that such a mechanism underlies the anti-inflammatory [49] and anti-obesity [50] effects in response to SCFA in the gut; however, there remains a large degree of heterogeneity in relevant findings and further research in this field is warranted [51]. Nonetheless, clinical studies in patients with type-2 diabetes have implicated lower levels of butyrogenic fecal microbiota, including *Faecalibacterium*, and gut microbiome dysbiosis [52], which may present relevance to individuals with PWS due to its increased prevalence in PWS as a comorbidity. *Leptotrichia* was also found to be enriched among the salivary microbiota post-probiotic treatment and existing literature has identified the genus as a part of the normal flora of the human oral cavity. In our study, we found that the relative abundance of *Leptotrichia* is positively correlated with the biosynthesis of N-glycans, neomycin metabolism, and negatively correlated with *staphylococcus aureus* infection post-BL-11 treatment. As past studies have suggested the importance of glycan expression in the bidirectional microbiota-host and inter-microbiota interactions within the oral microbiome for promoting host oral health and defense [53], these findings may suggest that the increase in *Leptotrichia* post-BL-11 intervention is a beneficial alteration to individuals with PWS in preventing oral infections from pathogenic species. Similarly, the analysis of predicted functional pathways from the salivary metagenome indicated that salivary *Bifidobacterium* was found to be significantly positively correlated with vitamin C metabolism and degradation of polycyclic aromatic hydrocarbons (PAH). Vitamin C is an antioxidant and has been suggested to promote host defense against periodontal diseases and promote general tooth and gingivae health [54]. Moreover, the positive correlation between *Bifidobacterium* abundance and degradation of PAH may suggest a role of *Bifidobacterium* in the oral metabolism and clearance of PAH. PAH have been characterized as pervasive environmental and dietary toxicants and carcinogens [55]. Existing literature has suggested that the toxicity of PAH is associated with its estrogenicity within the human colon following biotransformation of unabsorbed PAH due to colonic microbiota [56]. Given the findings within the current study, the positive correlation between PAH degradation and *Bifidobacterium* relative abundance observed in oral saliva samples is suggestive of the potential for BL-11 supplementation to decrease the levels of unabsorbed PAH reaching the colon, thereby reducing the likelihood of PAH-associated toxicity in subjects receiving the BL-11 probiotic. Thus, such findings represent post-probiotic treatment signatures of compositional alterations in the oral microbiota of individuals with PWS that present relevance to enhance host anti-inflammatory responses, contribute to interactions between microbiota, improve host immune defense against pathogenic microbes, and facilitate toxin degradation, though the causal relationship for such microbial functions remains to be validated in future studies.

Taken together, we find that oral BL-11 supplementation can induce specific favorable compositional changes in the salivary microbiota of individuals with PWS following a 12-week interventional period. We further propose that these specific salivary microbiota signatures may represent valuable features in the evaluation of probiotic treatment response as well as the early diagnosis of stunted anthropometric growth, social behavior deficits, and presence of comorbid ASD features in children with PWS. Additionally, due to the non-invasive nature of the sample collection, salivary microbiota sampling is likely preferred over fecal microbiota sampling, provided that the application of such a technique can demonstrate sufficient sensitivity and specificity in classification through future studies. However, due to the number of dropouts, small sample size due to limited saliva sample collection, and a homogenously Chinese study population, the results from the present study should be interpreted with caution and the generalizability of our findings should be carefully evaluated. We hope that the findings of this study could shed light on the complex interactions between the salivary microbiome and the effects of the probiotic strain, as well as changes in aberrant behaviors and associated autism symptoms observed in individuals with PWS in response to probiotic supplementation. Furthermore, given the observed influences on the salivary microbiota following oral supplementation of the BL-11 probiotic in a powder format, it is of interest to assess the potential for further research and development of novel routes of administration for oral-use probiotics.

## Supplementary Information

Below is the link to the electronic supplementary material.

Supplementary file1 (EPS 1428 KB)

Supplementary file2 (EPS 1222 KB)

Supplementary file3 (EPS 1412 KB)

## Data Availability

The data presented in this study are openly available in the Sequence Read Archive (SRA) database of The National Center for Biotechnology Information at https://www.ncbi.nlm.nih.gov/bioproject/643297, with accession number PRJNA643297.
